# Preclinical characterization of 3p-C-DEPA_-NCS_ and 3p-C-DEPA_-TFP_-PEG_4_ as potential Actinium-225 bifunctional chelators using DOTA_-NCS_ and macropa_-NCS_ as benchmarks

**DOI:** 10.1186/s41181-025-00408-w

**Published:** 2025-12-22

**Authors:** Jessica Pougoue Ketchemen, Stephen Ahenkorah, Emmanuel Nwangele, Siphelele Siphesihle Pearl Malaza, Maarten Ooms, Thomas Cardinaels, Simon Leekens, Frederik Cleeren, Humphrey Fonge

**Affiliations:** 1 Faculté de Pharmacie, Université Laval, Québec, QC Canada; 2 Axe Oncologie, Centre de Recherche du CHU de Québec-Université Laval, Québec, QC G1J 5B3 Canada; 3 Nuclear Medical Applications Institute, Belgian Nuclear Research Center (SCK CEN), Mol, Belgium; 4 Radiopharmaceutical Research, Department of Pharmaceutical and Pharmacological Sciences, University of Leuven (KU Leuven), Leuven, Belgium; 5 Department of Radiology, University of Iowa Health Care, 200 Hawkins Drive, Iowa City, IA USA; 6 Department of Chemistry, University of Leuven (KU Leuven), Leuven, Belgium; 7 Department of Medical Imaging, College of Medicine, University of Saskatchewan, Saskatoon, SK Canada

**Keywords:** Targeted alpha therapy, Actinium-225, Bifunctional chelators, 3p-*C-*DEPA, 3p-*C*-DEPA_-TFP_-PEG₄, Trastuzumab

## Abstract

**Background:**

Actinium-225 (^225^Ac) based targeted alpha therapies (TAT) have emerged as a promising strategy for the treatment of several cancer types due to its favourable decay properties, including high linear energy transfer and short particle range, which enable precise tumour targeting. However, there are limited bifunctional chelators (BFCs) available for ^225^Ac. In this study, we aim to evaluate the potential of DEPA-based chelators for ^225^Ac-labelling.

**Results:**

The BFCs 3p-*C*-DEPA-_NO2_, 3p-*C*-DEPA-_NCS_, and 3p-*C*-DEPA_-TFP_-PEG_4_ were synthesized with high yield (≥ 86%) and purity (> 96%). Excellent radiochemical conversions (RCCs) were achieved for [^225^Ac]Ac-3p-*C*-DEPA-_NO2_ across a range of concentrations (0.7- 13.4 µg) with high RCC’s (93.7 to 96.8%) after 1 h at room temperature. Stability studies demonstrated that over 95% of this ^225^Ac-labelled complex remained intact after 6 days in human serum. The HPLC and bioanalyzer analysis of the immunoconjugates 3p-*C*-DEPA_-TFP_-PEG_4_-trastuzumab, DOTA-trastuzumab, 3p-*C*-DEPA-trastuzumab and macropa-trastuzumab showed 98% purity with less than 2% impurities. A RCC of 94.6% was obtained for [^225^Ac]Ac-3p-*C*-DEPA-trastuzumab, 93.5% for [^225^Ac]Ac-3p-*C*-DEPA_-TFP_-PEG_4_-trastuzumab, 80.9% for [^225^Ac]Ac-DOTA-trastuzumab, and 96.5% for [^225^Ac]Ac-macropa-trastuzumab after 2 h incubation at 37 °C. In PBS, high stability of [^225^Ac]Ac-3p-*C*-DEPA_-TFP_-PEG_4_-trastuzumab was observed (91.3 ± 4.3%), which is comparable to that of [^225^Ac]Ac-macropa-trastuzumab (81.9 ± 5.6%). In contrast, [^225^Ac]Ac-3p-*C*-DEPA-trastuzumab and [^225^Ac]Ac-DOTA-trastuzumab were less stable in PBS with only 48.3 ± 1.2% and 60.1 ± 0.6% intact tracer left after 10 d. There were no major significant differences between the biodistribution profile of [^225^Ac]Ac-3p-C-DEPA-trastuzumab, [^225^Ac]Ac-3p-*C*-DEPA_-TFP_-PEG_4_-trastuzumab, [^225^Ac]Ac-DOTA-trastuzumab and [^225^Ac]Ac-macropa-trastuzumab in all organs of interest (*p* > 0.05 for all organs).

**Conclusions:**

3p-*C*-DEPA_-TFP_-PEG₄ demonstrated excellent potential as a bifunctional chelator for ^225^Ac, showing high radiolabelling efficiency under mild conditions and outstanding in vitro stability of the resulting ^225^Ac-labelled bioconjugate. Further preclinical studies are warranted to validate its therapeutic potential.

**Supplementary Information:**

The online version contains supplementary material available at 10.1186/s41181-025-00408-w.

## Background

Alpha-particle (α-particle) emitting radioisotopes have emerged as particularly promising agents for the selective eradication of cancer cells (Pougoue Ketchemen et al. 2024a). Due to their high linear energy transfer (LET) and short path length in biological tissues, α-particles are capable of depositing large amounts of energy over a short distance. This confers high cytotoxicity specifically to targeted tumour cells while minimizing collateral damage to surrounding healthy tissue. Importantly, the high LET of α-particles is more effective in inducing lethal DNA double-strand breaks compared to the lower LET of beta-minus particles (β^−^-particles) (Brechbiel 2007; Liberal, et al. 2020; Seidl 2014; Yang et al. 2022).

Several α-emitting radioisotopes, including ^225^Ac, have demonstrated superior therapeutic potential in both preclinical and clinical settings when compared with their β^−^-emitting counterparts such as Yttrium-90 (^90^Y) and Lutetium-177 (^177^Lu) (Ballal et al. 2020; Kratochwil et al. 2014; Kratochwil,, et al. 2016). Among α-emitting radioisotopes, ^225^Ac (t_1/2_ = 9.9 d, E_α_ = 5.8 MeV) is particularly notable due to its decay chain, which results in the emission of four α-particles, each contributing to its potent cytotoxic effect. Moreover, its relatively long half-life of 9.9 d is well-suited to long circulating biologicals such as monoclonal antibodies, whose pharmacokinetics align with this timeframe (Geerlings et al. 1993; Morgenstern et al. 2018).

The clinical success of targeted α-therapy in metastatic castration-resistant prostate cancer using [^225^Ac]Ac-PSMA-617 has significantly accelerated interest in the development of novel ^225^Ac-labelled radiopharmaceuticals for a wider spectrum of malignancies, including approaches based on ^225^Ac-labelled monoclonal antibodies (Kratochwil et al. 2016).

Efficient radiolabelling of ^225^Ac under mild conditions, combined with high kinetic inertness and thermodynamic stability of the resulting complex, is essential for the safe and effective application of targeted alpha therapy (TAT). These factors are critical to prevent in vivo dissociation of the radiometal, which can lead to off-target radiation exposure and dose-limiting toxicity (Nikula et al. 1999; Ahenkorah et al. 2021). The increasing clinical momentum behind TAT has intensified the development of new chelating agents specifically optimized for ^225^Ac, as the in vivo stability of the radiometal complex is a key determinant of therapeutic success. (Kim and Brechbiel 2012).

A variety of ^225^Ac-chelators have been reported, including EDTA, PEPA, CHX-DTPA, DTPA, DOTA, TETA, DOTMP, TETPA, and macropa. Among these, DOTA and macropa have shown the greatest promise in terms of practical applicability and coordination performance (Bidkar et al. 2024; Davis et al. 1999; Deal et al. 1999; Chappell et al. 2000; McDevitt et al. 2002). Derivatives of DOTA, a 12-membered macrocycle ligand with four nitrogen atoms and four pendant carboxylates, offer hepta/octa-dentate coordination geometry well-suited for many trivalent radiometals such as ⁶⁸Ga^3^⁺, ^111^In^3^⁺, and ^1^⁷⁷Lu^3^⁺. It has been successfully integrated into multiple FDA-approved radiopharmaceuticals, including [⁶⁸Ga]Ga-DOTATATE and [^1^⁷⁷Lu]Lu-DOTATATE, for both diagnostic and therapeutic use.

However, the use of DOTA for ^225^Ac is limited by several factors. Most notably, radiolabelling requires elevated temperatures (80–95 °C) to achieve high radiochemical yields, making it incompatible with heat-sensitive biomolecules such as monoclonal antibodies (Ahenkorah et al. 2022). From a clinical perspective, room-temperature labelling in under 20 min is preferred to streamline radiopharmaceutical preparation and to avoid radiolytic degradation of heat-sensitive targeting vectors (Price and Orvig 2014; Thiele and Wilson 2018). Additionally, the kinetic inertness of ^225^Ac-DOTA complexes has been challenged by several in vitro and in vivo studies, which report partial release of ^225^Ac from the chelator over time (Deal et al. 1999). This instability highlights DOTA’s limitations as a long-term carrier for ^225^Ac and underscores the need for chelators tailored to the unique coordination chemistry of large, low-charge-density ions. With an ionic radius of ~ 1.22 Å, Ac^3^⁺ forms weak electrostatic interactions with typical donor atoms, complicating efforts to achieve both strong binding and kinetic stability (Hu, et al. 2021).

Macropa (bp18c6), a 4,13-diaza-18-crown-6-macrocycle ligand with two pendant picolinate arms, has demonstrated strong chelation capabilities for ^225^Ac due to its high selectivity for large trivalent ions, particularly among the lanthanide and actinide series (Hu and Wilson 2022; Kadassery et al. 2022a). One of its major advantages over traditional chelators such as DOTA is its ability to form stable complexes with ^225^Ac within 5 min at room temperature, even at low micromolar concentrations (Kadassery et al. 2022a; Thiele et al. 2017). Macropa has also shown excellent in vitro and in vivo stability when conjugated to both small molecules and monoclonal antibodies (King et al. 2023). For example, Schatz et *al.* evaluated the efficacy of [^225^Ac]Ac-macropa-pelgifatamab in various cell-derived and patient-derived prostate cancer xenografts (Schatz et al. 2024).

Despite its favourable coordination properties, the macropa_-NCS_ derivative, commonly used for bioconjugation, suffers from limited shelf-life due to rapid degradation. This has prompted efforts to develop more chemically robust analogues. Recently, Kadassery et al*.* reported the synthesis of a more stable bifunctional macropa derivative, H_2_BZmacropa_-NCS_, via a five-step procedure (Kadassery et al. 2022b). However, the [^225^Ac]Ac-BZmacropa-GC33 conjugate (antibody codrituzumab (GC33), which targets the liver cancer biomarker glypican-3) showed only 55% stability in human serum, indicating that further optimization is needed to improve stability.

3p-*C*-DEPA_-NO2_ (1, 2- [(carboxymethyl)] [5-(4-nitrophenyl-1-[4, 7, 10-tris(carboxymethyl) -1, 4, 7, 10- tetraazacyclododecan-1-yl]pentan-2-yl)amino]acetic acid), is a decadentate ligand featuring ten electron donor atoms for complex formation. Its bifunctional derivative, 3p-*C*-DEPA_-NCS_, has been reported to rapidly chelate ^90^Y and ^177^Lu within 1 min, achieving radiolabelling efficiencies of 89% and 94%, respectively (Chong et al. 2015). However, to date, no data have been reported regarding its performance with ^225^Ac.

In this study, we aim to evaluate the potential of DEPA-based chelators for ^225^Ac-labelling. We first evaluated the radiolabelling efficiency and in vitro stability of the chelator 3p-*C*-DEPA-_NO₂_ with ^225^Ac. We then synthesized two bifunctional chelator (BFC) derivatives, 3p-*C*-DEPA-_NCS_ and 3p-*C*-DEPA-_TFP_-PEG_4_ and conjugated them to the monoclonal antibody trastuzumab. The radiochemical stability of the resulting ^225^Ac-conjugates was assessed, and finally, we conducted a biodistribution study to evaluate the in vivo behaviour. As a reference, macropa-_NCS_ and DOTA-_NCS_ were used in the conjugation and radiolabelling of trastuzumab to compare the stability and performance of these established chelators with the newly synthesized BFCs.

## Materials and methods

### General

All chemicals and solvents were purchased from commercial suppliers such as Sigma-Aldrich (Bornem, Belgium), Fluka (Bornem, Belgium), Fisher (Doornik, Belgium) and Acros Organics (Geel, Belgium) and were used without further purification. DOTA_-NCS_ was purchased from Macrocyclics, Inc (Plano, TX). Macropa_-NCS_ was synthesized as reported by Thiele et *al.* (Thiele et al. 2017) and 3p-*C*-DEPA_-TFP_-PEG_4_ was synthesized in Dr. Cleeren’s laboratory and analyzed using Nuclear Magnetic Resonance Spectroscopy (^1^H-NMR), High performance Liquid Chromatography (HPLC) and liquid chromatography mass spectroscopy (LC–MS). Details of the synthesis and characterization of 3p-*C*-DEPA_-TFP_-PEG_4_ are provided in the supplementary material. ^1^H-NMR was carried out in dimethyl sulfoxide (DMSO-d_6_). 3p-C-DEPA-TFP-PEG4 was purified using preparative HPLC using an X-bridge C-18, 4.6 X 250 mm column and the purified compound was analyzed by LC–MS using a Waters X-Bridge C-18 (3.5 µm, 3.0 × 100 mm) column coupled to a Waters e-2695 Separations Module, a Waters 2998 PDA UV Detector (λ = 210–400 nm) and Waters Acquity ESI QDa (negative mode, 100–1200 Da) system. The data was analysed using Waters’ Empower 3 software.

Refer to Kang et al*.* for details regarding the synthesis of 3p-*C*-DEPA_-NO2_ and 3p-*C*-DEPA_-NCS_ (Kang et al. 2015). All water was deionized and passed through Millipore water purification system until a resistivity of 18 MΩ·cm was achieved. Trastuzumab (RRID: AB_3112050) was of research grade and was purchased from Ichorbio (catalogue number: ICH4013, Oxford, UK). ^225^Ac for bioconjugate labelling was supplied by the Isotope Program within the Office of Nuclear Physics in the Department of Energy’s Office of Science (Oak Ridge National Laboratory, TN, USA).

Female Balb/C mice (RRID: IMSR_CRL:028) (n = 3/group) were purchased from Charles River (Saint-Constant, QC, CA), were at least 6 weeks of age and were used for biodistribution studies.

1. **Conjugation of bifunctional chelators to trastuzumab**.

Trastuzumab was conjugated with BFCs following lab SOPs (Ketchemen et al. 2023; Pougoue Ketchemen et al. 2024b; Tikum 2024). Briefly, a 15-mol excess of a 20 mg/mL of either, 3p-*C*-DEPA_-NCS_, 3p-*C*-DEPA_-TFP_-PEG_4_, or DOTA_-NCS_ in DMSO solution was incubated with trastuzumab in 0.1 M Na_2_CO_3_ pH 9, for 90 min at 37 °C with constant shaking. For macropa_-NCS_ conjugation (15 mol excess), the reaction was carried out in 0.1 M NaHCO_3_ and 0.15 M NaCl pH 9, for 18 h at 4 °C. The unreacted BFC was removed by centrifugation using Amicon Ultra-10 k (Burlington, MA) filters. 3p-*C*-DEPA-trastuzumab, 3p-*C*-DEPA_-TFP_-PEG_4_-trastuzumab, DOTA-trastuzumab, or macropa-trastuzumab, was buffer exchanged to PBS and concentrated with Amicon Ultra centrifugal filters which afforded > 96% purity. The purified conjugates were stored at -80 °C before labelling. The purity of the respective conjugates was performed using a size-exclusion HPLC (SEC-HPLC) Waters 2487 Dual λ Absorbance Detector, XBridge® BEH 200 A SEC 3.5 µm, 7.8 × 150 nm column (Waters Corporation, Milford, MA) and an Agilent 2100 Bioanalyzer system (Agilent High Sensitivity Protein 250 Kit- catalogue# 5067–1575) following the manufacturer’s protocol. The number of BFC per trastuzumab molecule or chelator-to-antibody ratio (CAR) was determined by Matrix-assisted laser desorption/ionization- time of flight (MALDI-TOF) mass spectrometry.

2. **Radiochemistry of [**^**225**^**Ac]Ac-3p-*C*-DEPA**_**-NO2**_.

[^225^Ac]Ac(NO_3_)_3_ (0.5 M HNO_3_) was produced on-site at SCK CEN based on literature (Cassells 2021; Dekempeneer et al. 2020; McAlister and Horwitz 2018) and from Oak Ridge National Lab ORNL (Oak Ridge, TN, USA) containing trace amounts of ^227^Ac. All radiolabelling buffers were treated with Chelex 100 [sodium form (50–100 mesh, Sigma Aldrich)] for 15 min to remove trace metals. All solutions were degassed and filtered before use.

The radiolabelling experiments were performed by reacting 90–100 kBq of [^225^Ac]Ac(NO_3_)_3_ at 25, 40, 55 or 95 °C for 1 h (h) with different concentrations of 3p-*C*-DEPA_-NO2_ (0.7, 3.3, 6.7 and 13.4 µg) in 0.37 M TRIS buffer, pH 8.5, V = 300 µL) to yield [^225^Ac]Ac-3p-*C*-DEPA_-NO2_. The % radiochemical conversion (RCC) was evaluated by instant thin-layer liquid chromatography (iTLC-SG, Varian, Diegem, Belgium). iTLC-SG papers were developed in an elution chamber using acetonitrile: water (75/25 v/v) such that bound ^225^Ac and daughter radionuclides will migrate with the solvent front to the upper part of the iTLC strip, while unbound radionuclides will remain at the lower part where the mixture was originally spotted. Once the solvent front reached the top of the iTLC strip, it was removed from the mobile phase and cut into two (top and bottom). The activity of the upper and lower part of the iTLC strip was measured with a gamma counter (Wallac Wizard 1480, PerkinElmer, Waltham, MA) using the ^213^Bi-peak window (380–500 keV) after a time delay of 24 h to allow ^213^Bi to reach equilibrium with ^225^Ac as described (Miederer et al. 2008). The %RCC is calculated as activity at the top half divided by the total activity (top + bottom) × 100.

3. **Radiochemistry of immunoconjugates**.

3p-*C*-DEPA-trastuzumab, 3p-*C*-DEPA_-TFP_-PEG_4_-trastuzumab, DOTA-trastuzumab, and macropa-trastuzumab immunoconjugates were radiolabelled using [^225^Ac]Ac(NO_3_)_3_ (2.0 MBq) dissolved in 0.1 M Hydrochloric acid (Optima grade, Fisher Scientific) at a specific activity of 8–9.6 kBq/µg as reported (Solomon et al. 2020). About 10 µL of ascorbic acid (150 g/L) was added to prevent radiolysis in all the reactions containing 150 mM ammonium acetate pH 6. The pH of the reaction was determined by spotting 0.5–1 µL of the reaction mixture onto Hydrion pH paper (range, 5.0–9.0) (Sigma-Aldrich); pH of a typical reaction was 5.8–6.0. The incubation was done at 37 °C on a shaker at 700 RPM for 2 h. A small aliquot of either the radioimmunoconjugate (RIC) or free [^225^Ac]Ac(NO_3_)_3_ (0.8 µL) was spotted on a strip of instant thin-layer chromatography silica gel impregnated paper (iTLC-SG, Agilent Technologies) to determine the extent of incorporation of ^225^Ac onto the antibody using mobile phase of 20 mM sodium citrate (pH 5.2) or evaluate the radio-iTLC profile of free/unbound actinium [^225^Ac]Ac(NO₃)₃, respectively. Radio-SEC/HPLC was performed to confirm the purity of the RICs as described above.

4. **In vitro stability**.

The in vitro stability of [^225^Ac]Ac-3p-*C*-DEPA_-NO2_ radio-complex was evaluated in PBS and human serum (HS). After radiolabelling 3p-*C*-DEPA_-NO2_, the radio-complex was purified with a Sep-Pak C_18_ Light cartridge (Waters, Eschborn, Germany). Briefly, the Sep-Pak C_18_ Light cartridge was pre-conditioned with absolute ethanol (5 mL) followed by water (5 mL). The reaction mixture was loaded onto the cartridge and washed with 6–8 mL water to remove unbound ^225^Ac. The pure radio-complex was eluted with 0.2 mL absolute ethanol, and the volume was brought to 0.5 mL by diluting with 0.3 mL of 0.9% NaCl. Eighty µL of the purified radio-complex was added to a 1 mL vial containing either 420 µL of PBS or HS, and the solution was incubated at 37 °C under constant gentle shaking. To determine the percentage of intact [^225^Ac]Ac-3p-*C*-DEPA_-NO2_, 5 µL samples were taken for radio-iTLC analysis at selected times points (30, 60 min, 2 d, 3 d, and 6 d). Development of iTLC and counting of activity was performed as described above.

The in vitro stability of the radioimmunoconjugates [^225^Ac]Ac-DOTA-trastuzumab, [^225^Ac]Ac-3p-*C*-DEPA-trastuzumab, [^225^Ac]Ac-3p-*C*-DEPA_-TFP_-PEG_4_-trastuzumab, and [^225^Ac]Ac-macropa-trastuzumab was determined in PBS at 37 °C for 10 days (1, 2, 3, 4, 7 and 10 d). Each radioimmunoconjugate (RIC) was incubated at the respective incubation conditions to make a final concentration of ~ 400 kBq/500 µL. To analyse the purity, aliquots of 9 µL of each radioimmunoconjugate was drawn for radio-iTLC and analysed as described above.

5. **In vivo biodistribution of radioimmunoconjugates**.

Biodistribution of [^225^Ac]Ac-3p-*C*-DEPA-trastuzumab, [^225^Ac]Ac-3p-*C*-DEPA_-TFP_-PEG_4_-trastuzumab, [^225^Ac]Ac-DOTA-trastuzumab, and [^225^Ac]Ac-macropa-trastuzumab was studied in healthy Balb/C mice (n = 3/group). The mice were housed under standard conditions in approved facilities with 12 h light/dark cycles and given food and water ad libitum throughout the duration of the studies following a tail vein injection of 11.1 kBq of each radiolabelled construct. Mice were sacrificed at 48 h post injection (p.i.), and the activity in organs was measured using gamma counters (^213^Bi-peak window (380–500 keV),Wallac Wizard 1480, PerkinElmer, and Hidex automatic gamma counter, 15–2000 keV, HIDEX, Finland) and expressed as the % injected activity per gram of the organ (%IA/g).

### Statistical analysis

All data were expressed as the mean ± standard error of mean (SEM). A two-way ANOVA with the Geisser-Greenhouse correction and Tukey’s multiple comparisons test was used to determine the statistical significance between the different mice groups. All graphs or figures were analysed using GraphPad Prism Version 10 (RRID:SCR_002798).

## Results

### Synthesis of 3p-***C***-DEPA, 3p-C-DEPA_-NCS_, and 3p-***C***-DEPA_-TFP_-PEG_4_

**Fig. 1:**
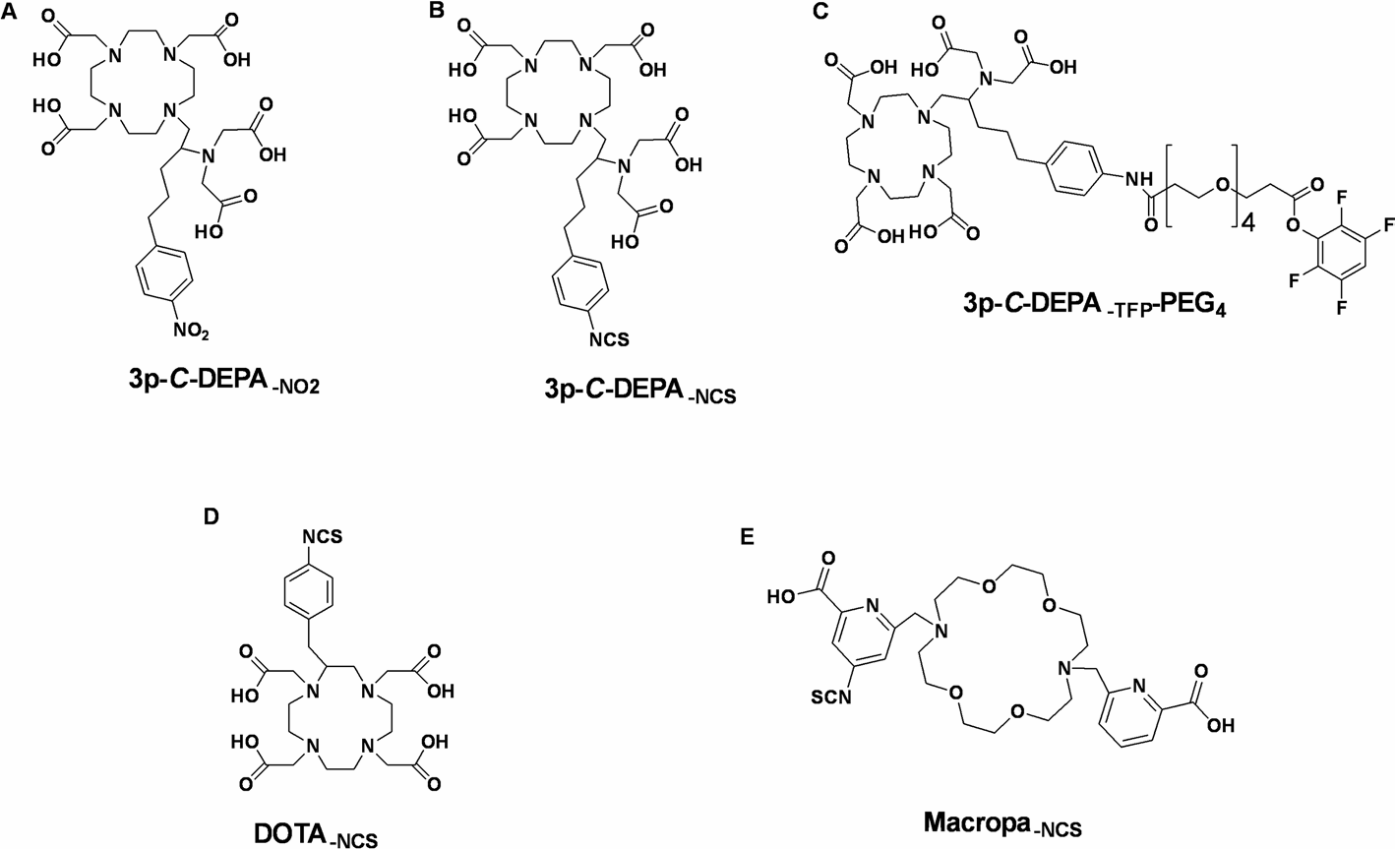
Chemical structures of (**A**) 3p-*C*-DEPA_-NO2_; (**B**) 3p-*C*-DEPA_-NCS_; (**C**) 3p-*C*-DEPA_TFP_-PEG_4_; coordination numbers = 10. (**D**) DOTA_-NCS_; coordination number = 8; and (**E**) macropa_-NCS_; coordination number = 10

Since 3p-*C*-DEPA is not commercially available, it was synthesized following a strategy involving the formation of an N,N′-bisubstituted-β-amino iodide intermediate and subsequent nucleophilic ring opening of an aziridinium ion, as previously described (Chong et al. 2015). Briefly, β-iodoamine was subjected to intramolecular rearrangement to form the aziridinium ion, which was then regioselectively opened via an SN_2_ mechanism using tri-tert-butyl 2,2′,2″-(1,4,7,10-tetraazacyclododecane-1,4,7-triyl)triacetate. This yielded the protected chelator 3p-*C*-DEPA-(*t*Bu)_5_ in an isolated yield of 86% and a purity exceeding 95%. The tert-butyl protecting groups were subsequently removed by treatment with trifluoroacetic acid (TFA), affording deprotected 3p-*C*-DEPA at > 96% purity. 3p-*C*-DEPA_-NO2_ (Fig. 1A) was used directly in initial radiolabelling and in vitro stability studies.

To enable conjugation of 3p-*C*-DEPA to a targeting vector such as trastuzumab, the chelator was further functionalized to introduce reactive groups capable of coupling to lysine residues. Two linker strategies were explored: isothiocyanate derivatization (Fig. 1B), as previously reported by Kang et al. (Kang et al. 2015), and PEG₄-TFP ester conjugation, a tetrafluorophenyl-activated ester linker. In the PEG₄-TFP approach, 3p-*C*-DEPA_-TFP_-PEG_4_ (Fig. 1C) was synthesized by the controlled, dropwise addition of 1.5 molar equivalents of TFP-PEG₄-TFP to 3p-*C*-DEPA-_NH₂_(tBu)₅. The chemical structures of DOTA_-NCS_ and macropa_-NCS_ are shown in Fig. 1D and E, respectively.

The reaction mixture of 3p-*C*-DEPA_-TFP_-PEG_4_ was purified using preparative HPLC, and the tert-butyl protecting groups were removed by treatment with TFA. The final product, 3p-*C*-DEPA-_TFP_-PEG_4_, was characterized by ^1^H-NMR, HPLC, and LC-HRMS, confirming > 95% chemical purity (Supplementary Fig. 1A-C). ^1^H NMR (300 MHz, DMSO-*d*_*6*_) δ 9.86 (s, 1H), 7.97–7.88 (m, 1H), 7.50 (d, ^*2*^* J* = 9 Hz, 2H), 7.12 (d, ^*2*^* J* = 9 Hz, 2H), 4.20—4.23 (m, 4H), 3.89—3.68 (coalesce signals, 28H), 3.18- 2.99 (coalesce signals, 10H), 2.43 (br t, 2H) 2.28 (s, 2H), 1.82 (s, 2H), 1.56—1.40 (coalesce signals, 10H), 1.23 (s, 6H), 0.8- 0.83 (m, 2H). The following m/z value was observed after deprotection; 532.37 (calculated for C_47_H_66_F_4_N_6_O_17_ [M + 2H]^2+^; 532.23).

### Radiolabelling and characterization of [.^225^Ac]Ac-3p-***C***-DEPA_-NO2_

**Fig. 2:**
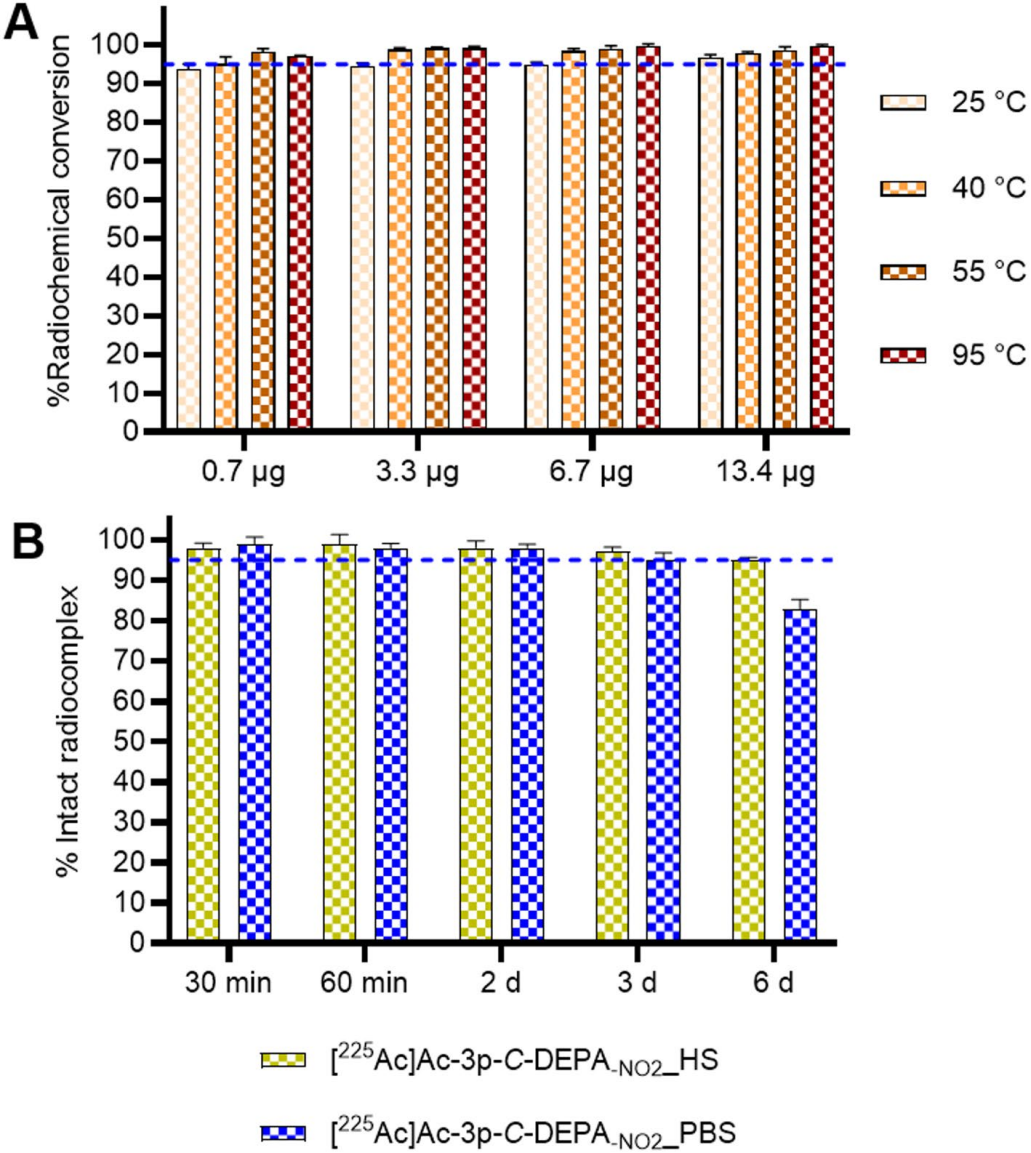
Radiolabelling and characterization of [^225^Ac]Ac-3p-*C*-DEPA_-NO2_. (**A**) Radiochemical conversions obtained for 3p-*C*-DEPA_-NO2_ with [^225^Ac]Ac(NO_3_)_3_. Ligand concentration of 0.7, 3.3, 6.7 and 13.4 µg (0.37 M Tris buffer, pH 8.5) with 90–100 kBq [^225^Ac]Ac(NO_3_)_3_ for 1 h, at 25, 40, 55 and 95 °C (n = 3). (**B**) In vitro stability studies in PBS and human serum for [^225^Ac]Ac-3p-*C*-DEPA_-NO2_. Stability was studied up to 6 days in triplicates (n = 3) at 37 °C. Blue line is inserted to indicate an intact radiocomplex of 95%

3p-*C*-DEPA-_NO₂_ demonstrated efficient radiolabelling with ^225^Ac under mild conditions. At a ligand concentration of 0.7 µg, rapid chelation was achieved within 1 h, with radiochemical conversion (RCC) values of 93.7 ± 1.4%, 95.1 ± 1.8%, and 98.2 ± 0.9% at 25 °C, 40 °C, and 55 °C, respectively. Further increases in ligand concentration (3.3–13.4 µg) and reaction temperature (40–95 °C) resulted in consistently high RCC values exceeding 97% under all tested conditions (Fig. 2A).

Given the favourable radiolabelling kinetics, we evaluated the in vitro stability of [^225^Ac]Ac-3p-*C*-DEPA-_NO₂_ over a six-day period (Fig. 2B). The radio-complex exhibited excellent stability in human serum, retaining > 95% of intact complex throughout the six-day incubation. In PBS, stability was also maintained over the first three days, with > 95% of intact radio-complex observed. By day six, 83.0 ± 4.1% of the radio-complex remained intact in PBS, indicating partial degradation under these conditions.

### Conjugation and characterization of immunoconjugates

**Fig. 3:**
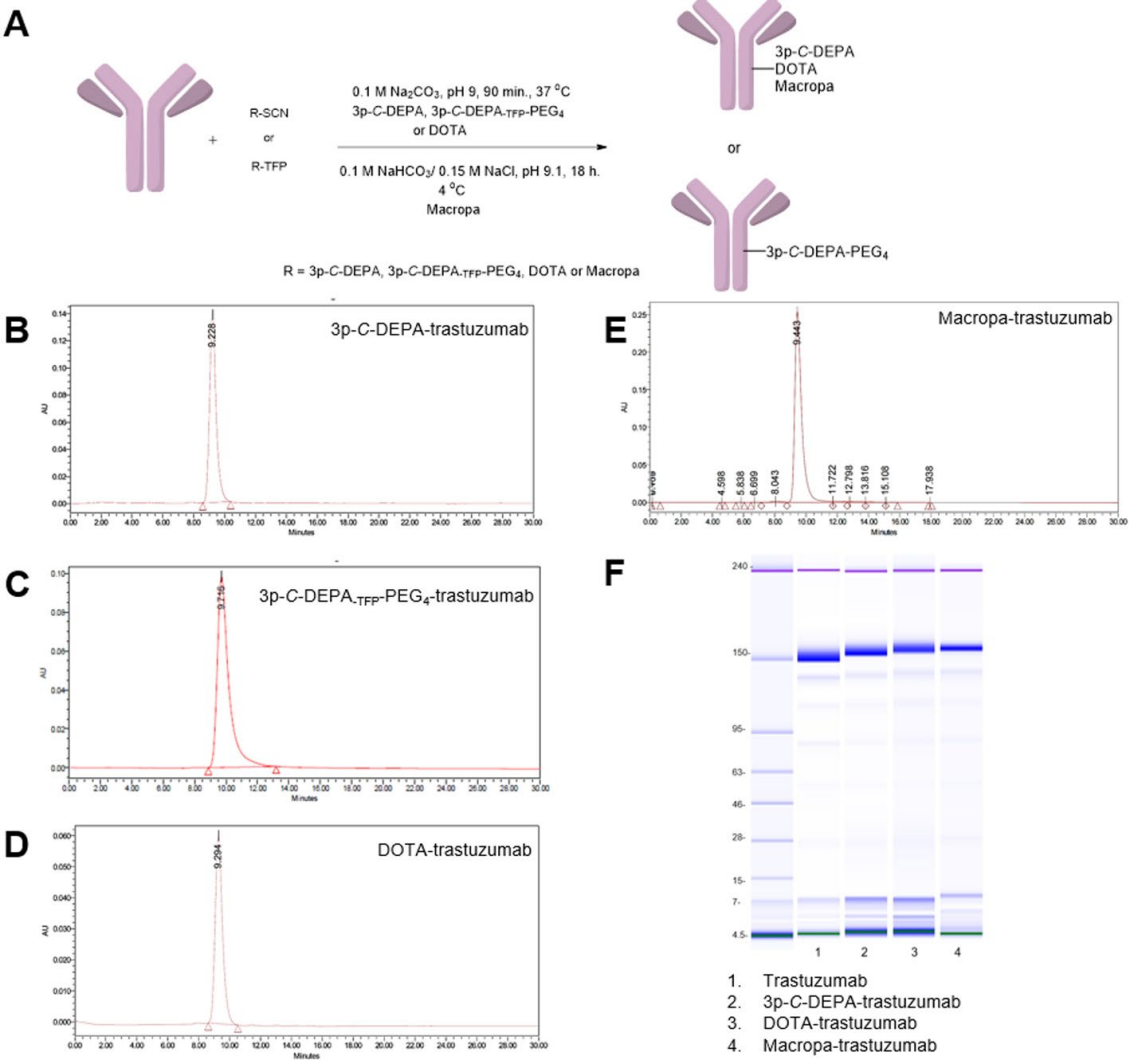
Schematic for the synthesis of trastuzumab conjugates and their characterization. (**A**) Chemical synthesis of 3p-*C*-DEPA-trastuzumab, 3p-*C*-DEPA_-TFP_-PEG_4_-trastuzumab, DOTA-trastuzumab, and macropa-trastuzumab. SEC-HPLC of (**B**) 3p-*C*-DEPA-trastuzumab, (**C**) 3p-*C*-DEPA_-TFP_-PEG_4_-trastuzumab, (**D**) DOTA-trastuzumab, and (**E**) macropa-trastuzumab (**F**) Bioanalyzer microfluidic electrophoresis of immunoconjugates

Encouraged by the initial radiolabelling studies with ^225^Ac using 3p-*C*-DEPA_-NO2_, the bifunctional chelators 3p-*C*-DEPA_-NCS_ and 3p-*C*-DEPA_-TFP_-PEG_4_ were conjugated (non-site specifically) to trastuzumab and compared with DOTA_-NCS_ and macropa_-NCS_-derivatized trastuzumab (Fig. 3A). Conjugation of trastuzumab with 3p-*C*-DEPA_-NCS_, 3p-*C*-DEPA_-TFP_-PEG_4_, DOTA_-NCS_, and macropa_-NCS_ yielded immunoconjugates 3p-*C*-DEPA-trastuzumab, 3p-*C*-DEPA_-TFP_-PEG_4_-trastuzumab, DOTA-trastuzumab, and macropa-trastuzumab (Fig. 3A), respectively. The HPLC showed that all the immunoconjugates were at least 98% pure with less than 2% degradation or aggregates (Fig. 3B-E). The bioanalyzer microfluidic electrophoresis experiment confirmed the purity of the various immunoconjugates (Fig. 3F). MALDI-TOF revealed that the chelator-to-antibody ratio (CAR) of 3p-*C*-DEPA-trastuzumab, 3p-*C*-DEPA_-TFP_-PEG_4_-trastuzumab, DOTA-trastuzumab, and macropa-trastuzumab was 1.2, 2.0, 3.6 and 2.1, respectively (Supplementary Figure S2 and Supplementary Table S1).

### Radiolabelling and characterisation of radioimmunoconjugates

**Fig. 4:**
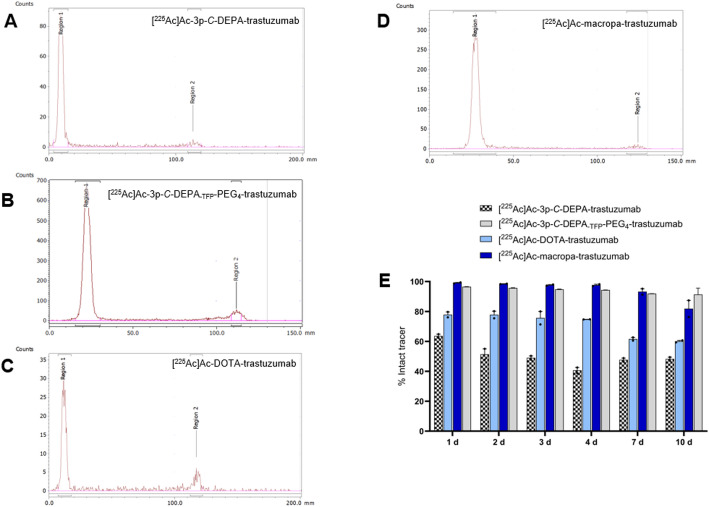
Characterization of radioimmunoconjugates. Radio-iTLC chromatograms of (**A**) [^225^Ac]Ac-3p-*C*-DEPA-trastuzumab, (**B**) [^225^Ac]Ac-3p-*C*-DEPA_-TFP_-PEG_4_-trastuzumab, (**C**) [^225^Ac]Ac-DOTA-trastuzumab, and (**D**) [^225^Ac]Ac-macropa-trastuzumab before purification. (**D**) In vitro stability of [^225^Ac]Ac-3p-*C*-DEPA-trastuzumab, [^225^Ac]Ac-3p-*C*-DEPA_-TFP_-PEG_4_-trastuzumab, [^225^Ac]Ac-DOTA-trastuzumab, and [^225^Ac]Ac-macropa-trastuzumab. (**E**) Stability was evaluated in PBS at 37 °C for 10 days (d) in duplicates

The immunoconjugates 3p-*C*-DEPA-trastuzumab, 3p-*C*-DEPA_-TFP_-PEG₄-trastuzumab, DOTA-trastuzumab, and macropa-trastuzumab were radiolabelled with [^225^Ac]Ac(NO₃)₃ at a specific activity of 8–9.6 kBq/μg. After 2 h of incubation at 37 °C, the RCC was 94.6% for [^225^Ac]Ac-3p-*C*-DEPA-trastuzumab, 93.5% for [^225^Ac]Ac-3p-*C*-DEPA_-TFP_-PEG₄-trastuzumab, 80.9% for [^225^Ac]Ac-DOTA-trastuzumab, and 96.5% for [^225^Ac]Ac-macropa-trastuzumab (Figs. 4A–4D). This was also confirmed from the ITLC of unbound actinium [^225^Ac]Ac(NO₃)₃ (Supplementary Figure S3).

The high RCCs observed for 3p-*C*-DEPA-, 3p-*C*-DEPA_-TFP_-PEG₄, and macropa-conjugated antibodies obviated the need for post-labelling purification. In contrast, [^225^Ac]Ac-DOTA-trastuzumab required purification by centrifugal filtration, yielding a final radiochemical purity of 95.9%. Radio-SEC/HPLC confirmed the radiochemical purity of all the RICs (Supplementary Figure S4).

The ^225^Ac-labelled immunoconjugates were evaluated for in vitro stability (PBS, 37 °C) over a 10-day period. Remarkably, [^225^Ac]Ac-3p-*C*-DEPA_-TFP_-PEG₄-trastuzumab exhibited the highest stability, retaining 91.3 ± 4.3% of the intact radiocomplex after 10 days. In comparison, [^225^Ac]Ac-macropa-trastuzumab maintained 81.9 ± 5.6% integrity, while [^225^Ac]Ac-DOTA-trastuzumab showed 60.1 ± 0.6% intact complex. The lowest stability was observed for [^225^Ac]Ac-3p-*C*-DEPA-trastuzumab, with only 48.3 ± 1.2% of the radiocomplex remaining intact at day 10 (Fig. 4E).

### Biodistribution of [^225^Ac]Ac-3p-***C***-DEPA-trastuzumab, [^225^Ac]Ac-DOTA-trastuzumab, [^225^Ac]Ac-macropa-trastuzumab, and [^225^Ac]Ac-3p-***C***-DEPA_-TFP_-PEG_4_-trastuzumab

Despite the relatively lower in vitro stability of [^225^Ac]Ac-3p-*C*-DEPA-trastuzumab in PBS, we proceeded to evaluate its in vivo biodistribution together with [^225^Ac]Ac-3p-*C*-DEPA_-TFP_-PEG_4_-trastuzumab in healthy Balb/C mice over a 48-h period, using [^225^Ac]Ac-DOTA-trastuzumab and [^225^Ac]Ac-macropa-trastuzumab as reference standards (Table S1). Generally, no significant differences were observed in the biodistribution profiles across major organs, with two exceptions.

In the large intestines, [^225^Ac]Ac-DOTA-trastuzumab showed lower accumulation (1.1 ± 0.1%IA/g) than [^225^Ac]Ac-macropa-trastuzumab (2.2 ± 0.1%IA/g, p = 0.04). Conversely, in the limbs, [^225^Ac]Ac-DOTA-trastuzumab (2.7 ± 0.2%IA/g, p = 0.05) showed lower accumulation than [^225^Ac]Ac-3p-*C*-DEPA_-TFP_-PEG_4_-trastuzumab (3.2 ± 0.3%IA/g).

**Fig. 5:**
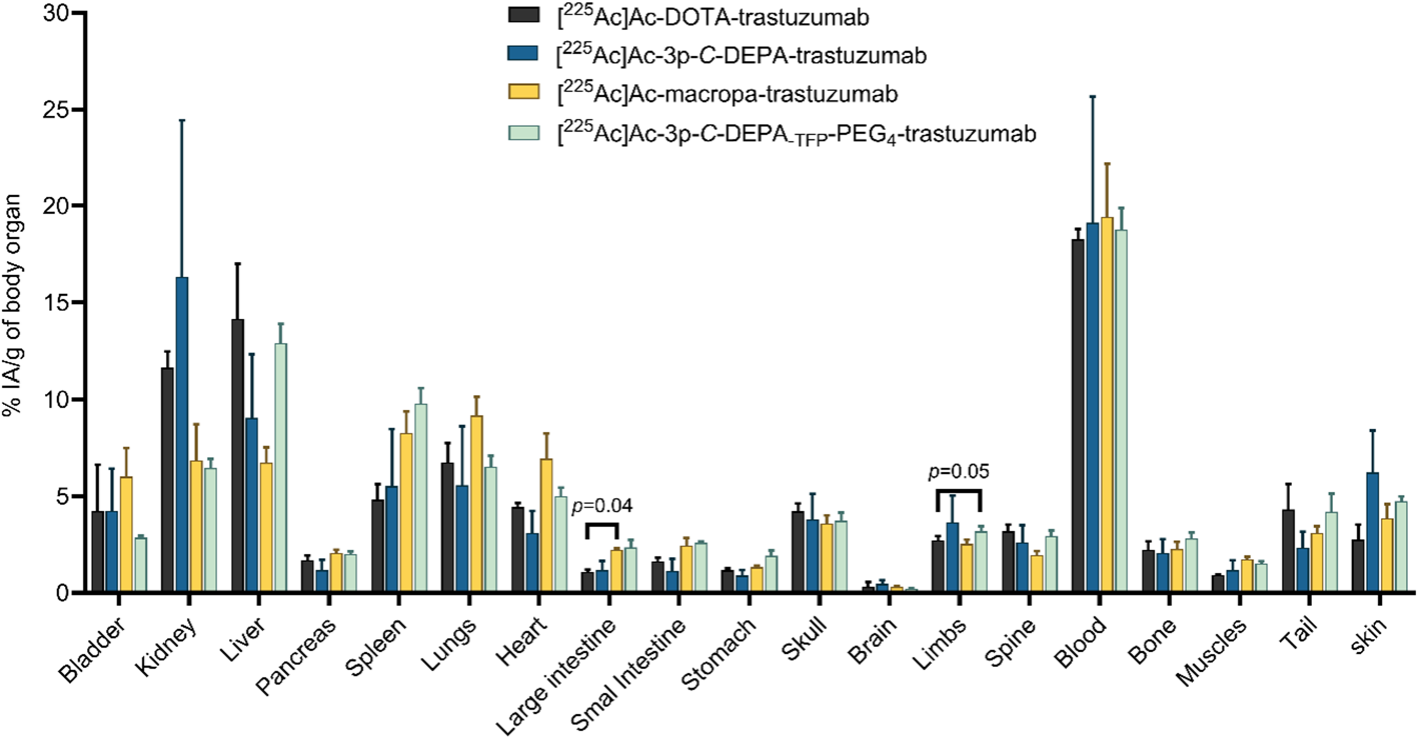
Selected organ biodistribution of [^225^Ac]Ac-3p-*C*-DEPA-trastuzumab, [^225^Ac]Ac-DOTA-trastuzumab, [^225^Ac]Ac-macropa-trastuzumab and [.^225^Ac]Ac-3p-*C*-DEPA_-TFP_-PEG_4_-trastuzumab at 48 h after a tail vein administration of 11.1 kBq activity (n = 3)

Consistent with expectations for radioimmunoconjugates, the highest activity was retained in the blood at 48 h post-injection, with values of 18.3 ± 0.5%IA/g for [^225^Ac]Ac-DOTA-trastuzumab, 19.1 ± 6.5%IA/g for [^225^Ac]Ac-3p-*C*-DEPA-trastuzumab, 19.4 ± 2.7%IA/g for [^225^Ac]Ac-macropa-trastuzumab and 18.8 ± 1.1%IA/g for [^225^Ac]Ac-3p-*C*-DEPA_-TFP_-PEG_4_-trastuzumab (Fig. 5, Supplementary Table S2).

## Discussion

^225^Ac is a highly promising radionuclide for TAT due to its favourable decay properties and potent cytotoxicity against cancer cells (Kratochwil et al. 2017; Ballal et al. 2019; Qin et al. 2020). Despite its therapeutic potential, the widespread clinical translation of ^225^Ac-based radiopharmaceuticals has been hindered by two major factors: limited availability of the isotope and the challenge of identifying suitable chelators for the large trivalent actinium ion (Ac^3^⁺). Although chelators such as DOTA, macropa, and macrodipa have been developed and studied extensively (Kadassery et al. 2022a; Thiele et al. 2017; Qin et al. 2020), each has limitations. DOTA remains the clinical standard for ^225^Ac coordination, but its slow radiolabelling kinetics, especially under mild conditions, pose significant challenges for heat-sensitive biomolecules such as monoclonal antibodies (Ahenkorah et al. 2021).

In this study, we investigated 3p-*C*-DEPA, a hybrid decadentate ligand combining structural features of DOTA and DTPA, as a potential alternative chelator for ^225^Ac. We successfully synthesized 3p-*C*-DEPA_-NO₂_ in high yield and purity, using a previously reported method for 3p-*C*-NETA (Ahenkorah et al. 2022; Kang et al. 2015). The presence of an additional acyclic iminodiacetic acid arm likely contributes to the faster complexation kinetics and improved radiolabelling performance observed with 3p-*C*-DEPA_-NO2_, compared to DOTA_-NCS_. Indeed, [^225^Ac]Ac-3p-*C*-DEPA_-NO₂_ demonstrated excellent radiolabelling efficiency (≥ 95% RCC) at room temperature at low chelator concentration, a significant advantage for radiolabelling heat-sensitive biomolecules whereas [^225^Ac]Ac-DOTA-trastuzumab showed a low RCC of 80.9%.

These findings align with previous work by Song et al., who noted the effectiveness of 3p-*C*-DEPA for large metal coordination due to its larger cavity size relative to DOTA (Song et al. 2011). In contrast, prior studies have shown that DOTA requires high temperatures (≥ 85 °C) to achieve efficient ^225^Ac complexation, with RCC values of only ~ 15% at 40 °C even after extended incubation (Ramogida et al. 2019). The ability of 3p-*C*-DEPA to rapidly and efficiently bind ^225^Ac at lower temperatures is thus a noteworthy improvement.

To assess the utility of 3p-*C*-DEPA in an antibody-based radiopharmaceutical, we synthesized two bifunctional derivatives: 3p-*C*-DEPA_-NCS_ and a novel PEGylated tetrafluorophenol ester (3p-*C*-DEPA-_TFP_-PEG₄). 3p-*C*-DEPA-_TFP_-PEG₄ is easy to synthesize starting from the reported 3p-C-DEPA-_NH₂_(tBu)₅ and demonstrate good stability when stored at -20 °C. Both bifunctional chelators were successfully conjugated to trastuzumab, resulting in the corresponding radioimmunoconjugates. Radiolabelling of [^225^Ac]Ac-3p-*C*-DEPA-trastuzumab and [^225^Ac]Ac-3p-C-DEPA_-TFP_-PEG₄-trastuzumab yielded high RCCs (94.6% and 93.5%, respectively), comparable to previous results using [^2^⁰^5^/^2^⁰⁶Bi]Bi-3p-*C*-DEPA-trastuzumab reported by Song et al. (Song et al. 2011).

However, in vitro stability studies in PBS revealed significant differences between the two constructs. [^225^Ac]Ac-3p-*C*-DEPA-trastuzumab showed notable degradation over 10 days (only 48.3 ± 1.2% remaining intact at day 10), likely due to radiolytic effects. Though this observation was surprising, it is reported in the literature that Cl^−^ (present in PBS) can undergo radiation-induced formation of hypochlorite ions which could potentially react with the enolized thiourea unit (Vugts et al. 2017). As 3p-*C*-DEPA is coupled to trastuzumab via a thiourea linker, this might be a reason for the observed instability. Remarkably, this effect was less observed with the macropa_-NCS_-conjugate which demonstrated high stability in PBS. To address this instability in PBS of [^225^Ac]Ac-3p-*C*-DEPA-trastuzumab, the PEGylated-TFP derivative was designed to form more stable amide bonds with lysine residues. Encouragingly, [^225^Ac]Ac-3p-*C*-DEPA_-TFP_-PEG₄-trastuzumab exhibited superior in vitro stability, maintaining > 90% integrity after 10 days in PBS.

In vivo biodistribution studies in healthy Balb/C mice showed that [^225^Ac]Ac-3p-*C*-DEPA-trastuzumab and [^225^Ac]Ac-3p-C-DEPA_-TFP_-PEG₄-trastuzumab exhibited a comparable organ distribution profile to both [^225^Ac]Ac-DOTA-trastuzumab and [^225^Ac]Ac-macropa-trastuzumab over 48 h. This indicates that the limited in vitro stability of [^225^Ac]Ac-3p-*C*-DEPA-trastuzumab in PBS could have limited consequences for the in vivo stability. Further studies will investigate this hypothesis.

Notably, liver uptake for [^225^Ac]Ac-3p-C-DEPA-trastuzumab (9.0 ± 3.3%IA/g), [^225^Ac]Ac-3p-C-DEPA_-TFP_-PEG₄-trastuzumab (12.9 ± 0.89%IA/g), and [^225^Ac]Ac-macropa-trastuzumab (6.7 ± 0.8%IA/g) were non-significantly different. This indicates that both the ^225^Ac-3p-C-DEPA, ^225^Ac-3p-C-DEPA_-TFP_-PEG₄ and ^225^Ac-macropa complexes exhibit comparable in vivo kinetic inertness. This distinction is particularly important given the known hepatotoxicity associated with free ^225^Ac (Deal et al. 1999; Price and Orvig 2014; Pruszynski et al. 2018). The high blood retention observed for all constructs reflects the long circulation time of intact antibody conjugates. However, studies in tumour-bearing mice are planned to assess their pharmacokinetics and tumour-targeting efficiency.

## Conclusion

This study demonstrates the promising potential of 3p-*C*-DEPA-based chelators for the development of ^225^Ac-labelled radiopharmaceuticals. Among the constructs evaluated, 3p-*C*-DEPA_-TFP_-PEG₄ stood out as a highly effective bifunctional chelator, achieving excellent radiolabelling efficiency under mild conditions and superior in vitro stability compared to DOTA analogues. Importantly, [^225^Ac]Ac-3p-*C*-DEPA_-TFP-_PEG₄-trastuzumab maintained > 90% integrity in PBS over 10 days, indicating strong resistance to radiolytic and hydrolytic degradation. Additionally, the biodistribution profile of 3p-*C*-DEPA-based conjugates was comparable to established chelators. These findings highlight 3p-*C*-DEPA_-TFP_-PEG₄ as a promising alternative to current ^225^Ac-bifunctional chelators, offering advantages in radiolabelling kinetics and stability. Further preclinical studies, including pharmacokinetic studies in mouse tumour models, therapeutic efficacy and long-term in vivo stability studies are warranted to confirm its utility in TAT applications.

## Supplementary Information

Below is the link to the electronic supplementary material.

Supplementary Material 1

## Data Availability

The datasets used and/or analysed during the current study are available from the corresponding authors Prof. Fonge and Prof. Cleeren on reasonable request.

## Abbreviations

BFC: Bifunctional chelators
DOTA: 1,4,7,10-Tetraazacyclododecane-1,4,7,10-tetraacetic acid
DTPA: Diethylenetriamine pentaacetic acid
EDTA: Ethylenediaminetetraacetic acid
FDA: Food and Drug Administration
HPLC: High performance liquid chromatography
HS: Human serum
ITLC: Instant thin-layer chromatography
LC-HRMS: Liquid chromatography- High resolution mass spectrometry
LET: Linear energy transfer
MALDI-TOF: Matrix-assisted laser desorption/ionization-time of flight
MS: Mass spectroscopy
p.i: Post injection
PBS: Phosphate-buffered saline
PSMA: Prostate specific membrane antigen
RCC: Radiochemical conversion
RIC: Radioimmunoconjugate
RPM: Revolutions per minutes
SEM: Standard error of mean
TAT: Targeted alpha therapies
TETA: Triethylenetetramine
TETPA: Triethylene tetramine pentaacetic acid
TFA: Trifuoroacetic acid
TFP: Tetrafluorophenol
