# Supplementary material for: Preclinical characterization of 3p-C-DEPA-NCS and 3p-C-DEPA-TFP-PEG4 as potential Actinium-225 bifunctional chelators using DOTA-NCS and macropa-NCS as benchmarks
Source: EJNMMI Radiopharm Chem. 2025 Dec 22;10:81. doi: 10.1186/s41181-025-00408-w (PMC12722590; doi:10.1186/s41181-025-00408-w)
Supplement: Supplementary file 1 — Supplementary Material 1 [file 41181_2025_408_MOESM1_ESM.docx]

***Preclinical characterization of 3p-C-DEPA_-NCS_ and 3p-C-DEPA_-TFP_-PEG_4_ as potential Actinium-225 bifunctional chelators using DOTA_-NCS_ and macropa_-NCS_ as benchmarks.***

*Jessica Pougoue Ketchemen^1,2‡^, Stephen Ahenkorah^3,4,5‡^, Emmanuel Nwangele^1,2^, Siphelele Siphesihle Pearl Malaza^4^, Maarten Ooms^3^, Thomas Cardinaels^6,7^, Simon Leekens^4^, Frederik Cleeren^4*^, Humphrey Fonge^1,2,8*^*

^1^Faculté de Pharmacie, Université Laval, Québec, QC, Canada

^2^Axe Oncologie, Centre de Recherche du CHU de Québec-Université Laval, Québec, QC, Canada

^3^Nuclear Medical Applications Institute, Belgian Nuclear Research Center (SCK CEN), Mol, Belgium.

^4^Radiopharmaceutical Research, Department of Pharmaceutical and Pharmacological Sciences, University of Leuven, Leuven, Belgium.

^5^Department of Radiology, University of Iowa Health Care, 200 Hawkins Drive, Iowa City, IA, USA

^6^Department of Chemistry, University of Leuven, Leuven, Belgium

^7^Flemish Institute for Technological Research (VITO), Materials and Chemistry Unit, Mol, Belgium

^8^Department of Medical Imaging, College of Medicine, University of Saskatchewan, Saskatoon, SK, Canada

**Supplementary information:**

**Chemicals**

Triethylamine was purchased from Sigma-Aldrich and used without purification. All HPLC grade solvents used were purchased from Merck and de-ionized water was generated in-house using a Millipore water purification system. Bis-TFP-PEG_4_ was purchased from MedChemExpress (Monmouth Junction, NJ).

**Synthesis, purification and characterization of 3p-C-DEPA_-TFP_-PEG_4_**

3p-C-DEPA-NH_2_ (^t^Bu_5_) was synthesized analogous to the method by Song *et al*. [1] 16.2 mg (1 molar eq.) of 3p-C-DEPA-NH2 (tBu)5 in 0.5 mL ACN was added to 1.5 molar eq. of TFP-PEG_4_-TFP (MedChemExpress, Monmouth Junction, USA) and 1.5 molar eq. of DIPEA in 0.5 mL ACN (drop-by-drop). The reaction was carried out at 60°C, for 48 h under continuous stirring. The resulting crude mixture was purified using preparative HPLC*.* 3p-*C*-DEPA-TFP(tBu)_5_ was redissolved in 2 mL 100% TFA and left to deprotect for 4.5 h under continuous stirring at room temperature followed by purification using preparative HPLC.

Preparative HPLC was performed using an Agilent 1100 Series HPLC system fitted with an X-Bridge C-18 (5 µm, 4.6 x 250 mm) column and injection volume of 10 – 15 µL. The analysis software was GINA star 5.8 (Elysia-Raytest). Mobile phase comprised of 0.1%TFA/deionized Water (solvent A) and 0.1%TFA/Acetonitrile (solvent B) at a flow rate of 1 mL/min over 30 minutes. To remove impurities, a gradient of 30 – 80% solvent B over 10 minutes followed by isocratic 10% solvent B over 5 minutes was used. Thereafter, isocratic 95% solvent B for 10 minutes to elute the desired compound. Subsequently, the mobile phase was set to a gradient from 95 – 30% solvent B for equilibration. The collected fractions were dried using a benchtop Labconco FreeZone 2.5 L freeze dryer at -84 ºC prior to characterization.

Nuclear magnetic resonance (NMR) spectra were recorded on a Bruker Ultrashield 300 Plus spectrometer (^1^H: 300 MHz, ^19^F{^1^H}:282 MHz) at ambient temperature. All chemical shifts are reported in the standard δ notation of parts per million using tetramethylsilane (TMS) as an internal standard and were referenced relative to the signal of the deuterated solvent, DMSO-*d_6_*. The J coupling constants are reported in Hertz (Hz). Spin multiplicities are reported in abbreviated conventional format (s = singlet, m = multiplet, d = doublet, br t = broad triplet). ^1^H NMR (300 MHz, DMSO-*d_6_*) δ 9.86 (s, 1H), 7.97-7.88 (m, 1H), 7.50 (d, *^2^J* =9 Hz, 2H) , 7.12 (d, *^2^J* =9 Hz, 2H) , 4.20 - 4.23 (m, 4H), 3.89 - 3.68 (coalesce signals, 28H), 3.18- 2.99 (coalesce signals, 10H), 2.43 (br t, 2H) 2.28 (s, 2H), 1.82 (s, 2H), 1.56 - 1.40 (coalesce signals, 10H), 1.23 (s, 6H), 0.8- 0.83 (m, 2H).

Purity and identity of the end-product was verified using LC-HRMS. LC-MS data was obtained using a Waters e-2695 Separations Module coupled to a Waters 2998 PDA UV Detector (λ =210-400 nm) and a Waters Acquity ESI QDa (negative mode, 100-1200 Da) system. The data was analysed using Waters’ Empower 3 software. The column used was an X-Bridge C-18 (3.5 µm, 3.0 x 100 mm). The mobile phase comprised 0.1% formic acid/deionized Water (solvent A) and 0.1% formic acid/Acetonitrile (solvent B) at a flow rate of 0.8 mL/min. The mobile phase was isocratic 5% solvent B for 3 minutes, a gradient of 5 – 95% solvent B over 10 minutes and isocratic 95% solvent B for a minute followed by 5% solvent B for 2 minutes. The following m/z value was observed after deprotection; 532.37 (calculated for C_47_H_66_F_4_N_6_O_17_ [M + 2H]^2+;^ 532.23).

**
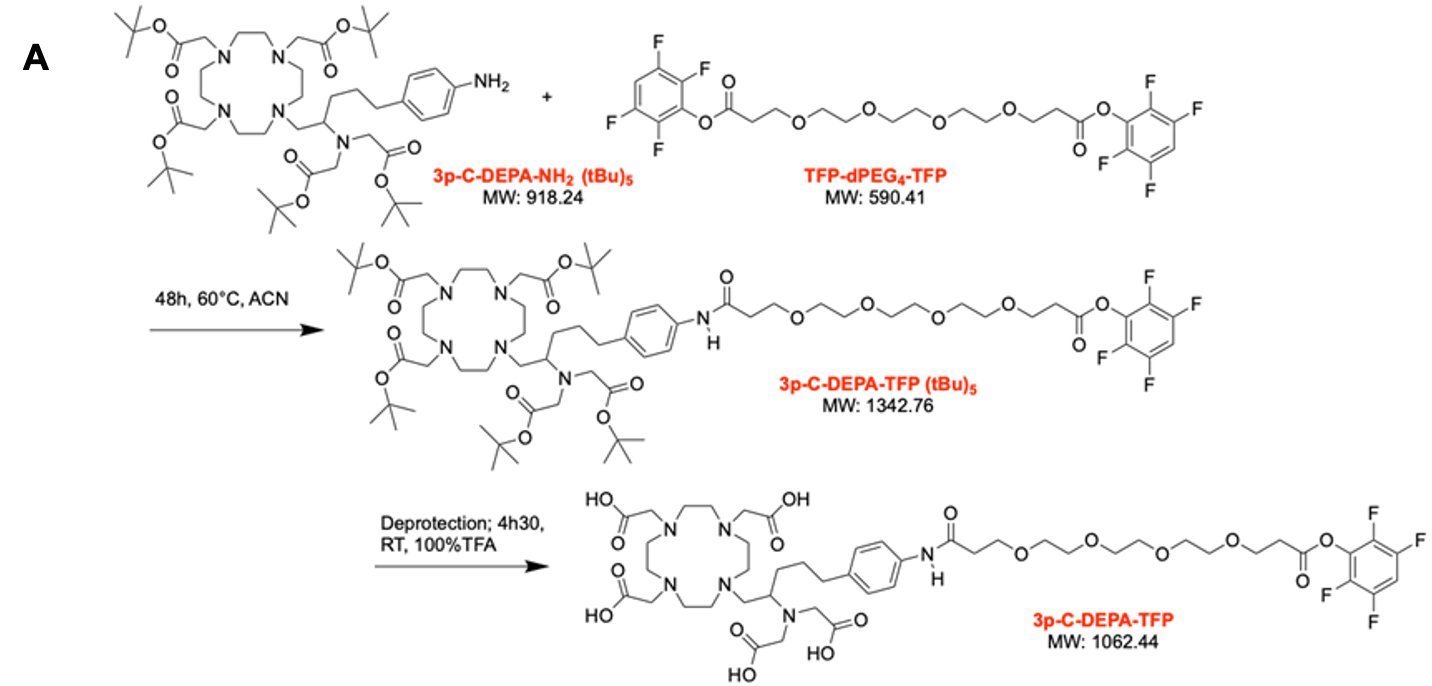
**

**
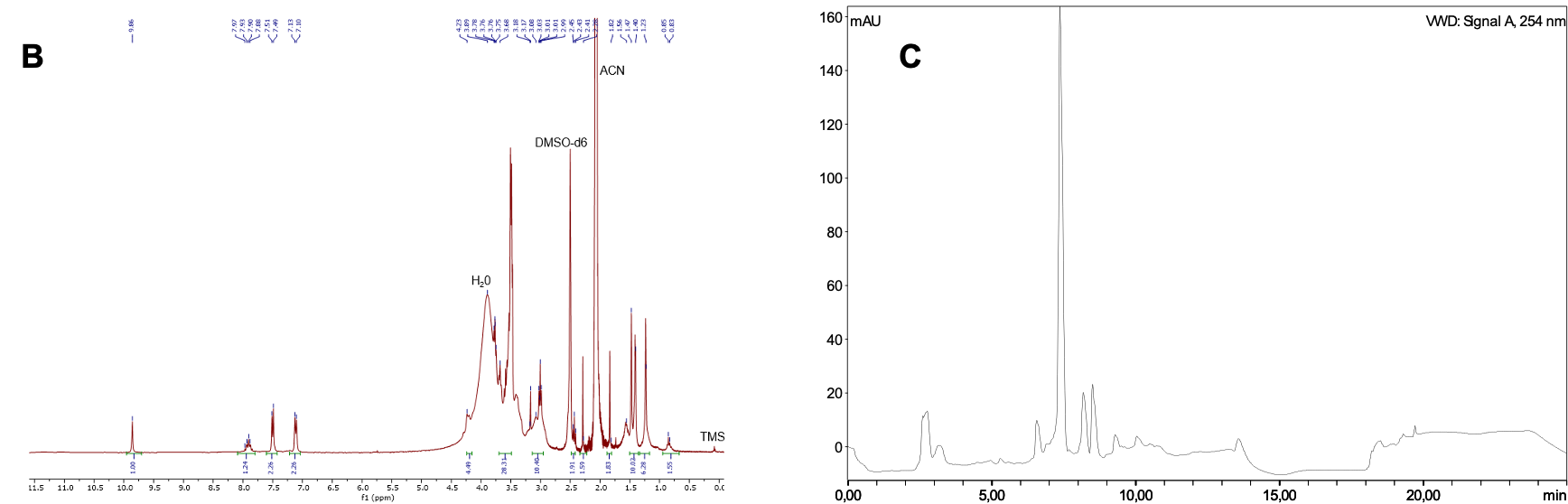
**

**Fig. S1:** Synthesis and analyses of 3p-*C*-DEPA_-TFP_-PEG_4_. (A) reaction scheme, (B) ^1^H-NMR spectrum of 3p-*C*-DEPA_-TFP_-PEG_4_ in DMSO-d6; (D) High Performance Liquid Chromatography (HPLC) of the crude reaction mixture of 3p-*C*-DEPA_-TFP_-PEG_4_ using X-Bridge C18 column (5 µm) 4.6 X 250 mm.


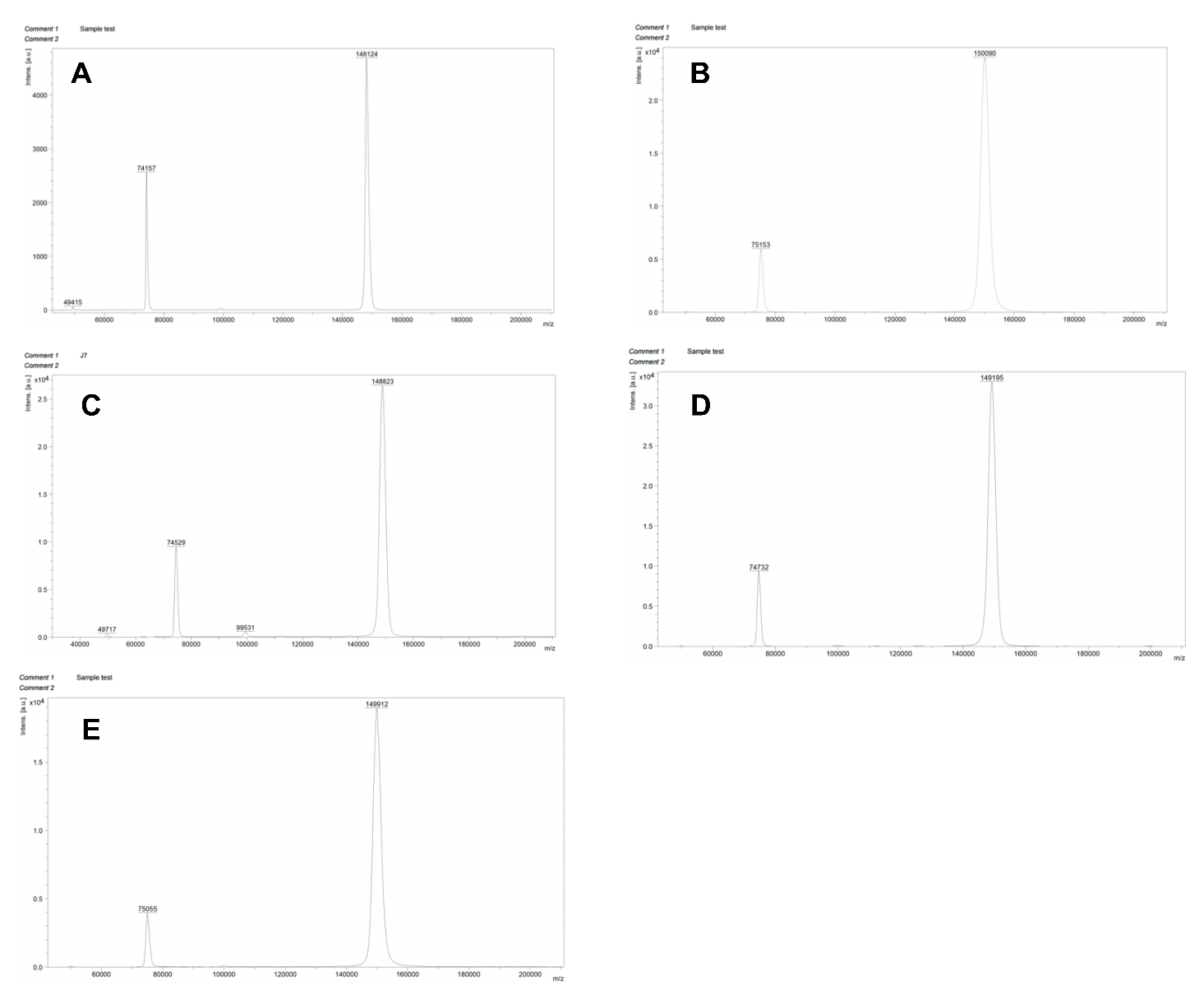


**Fig. S2:** Matrix-assisted laser desorption/ionization- Time of flight (MALDI-TOF) mass spectrometry of trastuzumab and its conjugates. (A) trastuzumab, and (B) DOTA-trastuzumab, (C) 3p-*C*-DEPA-trastuzumab, (D) macropa-trastuzumab, and (E) 3p-*C*-DEPA_-TFP_-PEG_4_-trastuzumab

**Table S1.** Chelator-to-antibody ratio (CAR) of trastuzumab immunoconjugates based on MALDI-TOF mass spectrometry.

| **Construct** | **3p-C-DEPA-trastuzumab** | **DOTA-trastuzumab** | **Macropa-trastuzumab** | **3p-C-DEPA-_TFP_-PEG_4_-trastuzumab** |
| --- | --- | --- | --- | --- |
| Chelator-to-antibody ratio (CAR) | 1.2 | 3.6 | 2.1 | 2.0 |


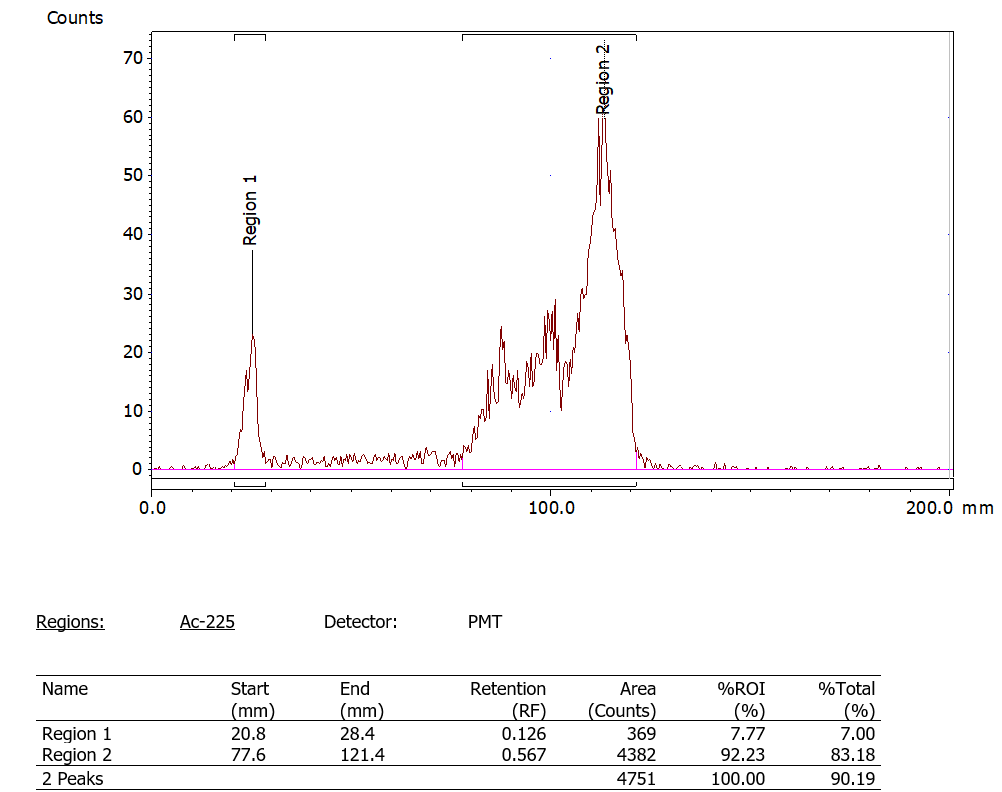


**Fig. S3**. Radio Instant thin layer chromatography (radio-iTLC) of free actinium nitrate [^225^Ac]AcNO_3_. More than 92% of free actinium (^225^Ac) is observed at the top of the ITLC strip.


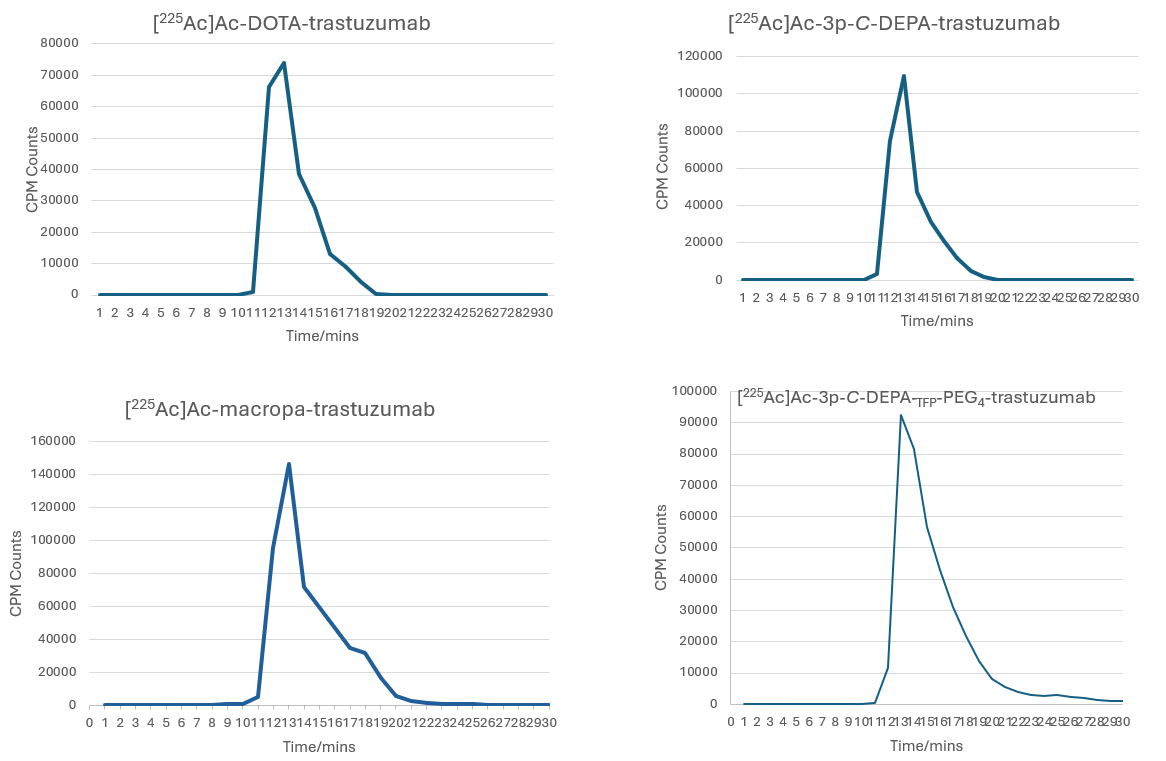


**Fig. S4**. Radio-SEC HPLC of radioimmunoconjugates (RIC). Each 50 kBq of radioimmunoconjugate (RIC) was injected in the radio-SEC HPLC for a 30 minute run. The activity of the samples collected into tubes were counted using the gamma counter and the results were then plotted with respect to time, confirming the purity of the RICs.

**Table S2:** Biodistribution of [^225^Ac]Ac-labeled antbody conjugates expressed as % injected activity per gram tissue (%IA/g)

| Organs % IA/g (mean ± SEM) | [^225^Ac]Ac-3p-*C*-DEPA-trastuzumab | [^225^Ac]Ac-DOTA-trastuzumab | [^225^Ac]Ac-macropa-trastuzumab | [^225^Ac]Ac-3p-*C*-DEPA-_TFP_-PEG_4_-trastuzumab |
| --- | --- | --- | --- | --- |
| Bladder | 4.2 ± 2.2 | 4.2 ± 2.4 | 6.0 ± 1.5 | 2.9 ± 0.1 |
| Kidney | 16.3 ± 8.1 | 11.6 ± 0.8 | 6.8 ± 1.9 | 6.5 ± 0.5 |
| Liver | 9.0 ± 3.3 | 14.1 ±2.9 | 6.7 ± 0.8 | 12.9 ± 1 |
| Pancreas | 1.2 ± 0.5 | 1.7 ± 0.2 | 2.1 ± 0.2 | 2.0 ± 0.2 |
| Spleen | 5.5 ± 3.0 | 4.8 ± 0.8 | 8.3 ± 1.1 | 9.8 ± 0.2 |
| Lungs | 5.6 ± 3.1 | 6.7 ±1 | 9.2 ± 1 | 6.5 ± 0.6 |
| Heart | 3.1 ± 1.1 | 4.4 ± 0.2 | 6.9 ± 1.3 | 5.0 ± 0.4 |
| Large intestine | 1.2 ± 0.5 | 1.1 ± 0.1 | 2.2 ± 0.1 | 2.4 ± 0.4 |
| Small intestine | 1.1 ± 0.6 | 1.6 ± 0.2 | 2.4 ± 0.4 | 2.6 ± 0.1 |
| Stomach | 0.9 ± 0.3 | 1.2 ± 0.1 | 1.3 ± 0.1 | 1.9 ± 0.3 |
| Skull | 3.8 ± 1.3 | 4.2 ± 0.4 | 3.6 ± 0.4 | 3.7 ± 0.4 |
| Brain | 0.5 ± 0.2 | 0.3 ± 0.3 | 0.3 ± 0.1 | 0.2 ± 0.01 |
| Limbs | 3.7 ± 1.4 | 2.7 ± 0.2 | 2.5 ± 0.2 | 3.2 ± 0.3 |
| Spine | 2.6 ± 0.9 | 3.2 ± 0.3 | 2 ± 0.2 | 2.9 ± 0.3 |
| Blood | 19.1 ± 6.5 | 18.3 ± 0.5 | 19.4 ± 2.8 | 18.8 ± 1.1 |
| Bone | 2.1 ± 0.7 | 2.2 ± 0.4 | 2.3 ± 0.4 | 2.8 ± 0.3 |
| Muscles | 1.2 ± 0.5 | 0.9 ± 0 | 1.7 ± 0.1 | 1.5 ± 0.1 |
| Tail | 2.3 ± 0.8 | 4.3 ± 1.3 | 3.1 ± 0.3 | 4.2 ± 0.9 |
| Skin | 6.2 ± 2.2 | 2.8 ± 0.8 | 3.8 ± 0.7 | 4.7 ± 0.2 |

Reference

1. Song HA, Kang CS, Baidoo KE, Milenic DE, Chen Y, Dai A, et al. Efficient bifunctional decadentate ligand 3p-C-DEPA for targeted alpha-radioimmunotherapy applications. Bioconjug Chem. 2011;22:1128-35. doi:10.1021/bc100586y.
